# A process for Decision-making after Pilot and feasibility Trials (ADePT): development following a feasibility study of a complex intervention for pelvic organ prolapse

**DOI:** 10.1186/1745-6215-14-353

**Published:** 2013-10-25

**Authors:** Carol Bugge, Brian Williams, Suzanne Hagen, Janet Logan, Cathryn Glazener, Stewart Pringle, Lesley Sinclair

**Affiliations:** 1 School of Nursing, Midwifery and Health, University of Stirling, Stirling FK9 4LA, Scotland; 2 Nursing, Midwifery and Allied Health Professions Research Unit, University of Stirling, Stirling FK9 4LA, Scotland; 3 Nursing, Midwifery and Allied Health Professions Research Unit, Glasgow Caledonian University, Cowcaddens Road, Glasgow G4 0BA, Scotland; 4 Health Services Research Unit, University of Aberdeen, Level 3, Health Sciences Building, Foresterhill, Aberdeen AB25 2ZD, Scotland; 5 Southern General Hospital, NHS Greater Glasgow and Clyde, 1345 Govan Road, Glasgow G51 4TF, Scotland; 6 School of Management, University of Stirling, Stirling FK9 4LA, Scotland

**Keywords:** Complex intervention, Pilot trial, Feasibility study, Methodology, Pelvic organ prolapse

## Abstract

**Background:**

Current Medical Research Council (MRC) guidance on complex interventions advocates pilot trials and feasibility studies as part of a phased approach to the development, testing, and evaluation of healthcare interventions. In this paper we discuss the results of a recent feasibility study and pilot trial for a randomized controlled trial (RCT) of pelvic floor muscle training for prolapse (ClinicalTrials.gov: NCT01136889). The ways in which researchers decide to respond to the results of feasibility work may have significant repercussions for both the nature and degree of tension between internal and external validity in a definitive trial.

**Methods:**

We used methodological issues to classify and analyze the problems that arose in the feasibility study. Four centers participated with the aim of randomizing 50 women. Women were eligible if they had prolapse of any type, of stage I to IV, and had a pessary successfully fitted. Postal questionnaires were administered at baseline, 6 months, and 7 months post-randomization. After identifying problems arising within the pilot study we then sought to locate potential solutions that might minimize the trade-off between a subsequent explanatory versus pragmatic trial.

**Results:**

The feasibility study pointed to significant potential problems in relation to participant recruitment, features of the intervention, acceptability of the intervention to participants, and outcome measurement. Finding minimal evidence to support our decision-making regarding the transition from feasibility work to a trial, we developed a systematic process (A process for Decision-making after Pilot and feasibility Trials (ADePT)) which we subsequently used as a guide. The process sought to: 1) encourage the systematic identification and appraisal of problems and potential solutions; 2) improve the transparency of decision-making processes; and 3) reveal the tensions that exist between pragmatic and explanatory choices.

**Conclusions:**

We have developed a process that may aid researchers in their attempt to identify the most appropriate solutions to problems identified within future pilot and feasibility RCTs. The process includes three key steps: a decision about the type of problem, the identification of all solutions (whether addressed within the intervention, trial design or clinical context), and a systematic appraisal of these solutions.

## Background

Over the past 15 years, there has been increasing formal guidance and a growing number of methodological papers, opinions, and commentaries published highlighting the need for greater attention to be paid to both the initial development of healthcare interventions and the design of their evaluation [1-3]. Such attention is understandable given both the increasing cost associated with delivering high quality trials [4], the number of interventions that fail to demonstrate effectiveness, and the difficulties in translating any demonstrable benefits into real-world contexts [5]. There have been suggestions that many of these translational issues may be ameliorated by addressing transferability to practice issues within earlier stages of intervention design [6-8].

Common recommendations include the need for thorough feasibility work to be conducted in order to pre-empt problems with the intervention itself, to support the implementation of a robust trial or other evaluative design, and for transferability to real-world practice. For example, current Medical Research Council (MRC) guidance on complex intervention evaluation advocates two distinct processes as part of a phased approach to the development, evaluation, and implementation of complex interventions in healthcare [1,2]. This includes feasibility studies (asking questions about whether the study can be done) and pilot trials (a miniature version of the main trial), which aim to test aspects of study design and processes for the implementation of a larger main trial in the future [2,9,10]. This increasingly detailed preparatory stage aims to maximize the likelihood of delivering a definitive trial that is high in terms of both internal validity (the scientific robustness of the trial) and external validity (its generalizability to routine real-world contexts). Simultaneously achieving both internal and external validity may be problematic; indeed, they may be in tension [11,12]. Consequently, two categories of trial have entered the methodological language [13,14]: explanatory trials, which focus on establishing efficacy (that is, can the intervention work?) with resultant high internal but low external validity; and pragmatic trials, which focus on effectiveness (that is, does the intervention work in real-world contexts?) with resultant lower internal but higher external validity.

Despite the recognized importance of feasibility and pilot work, two problems exist. First, several authors have suggested that the results of most contemporary pilot trials or feasibility studies are published with a focus on outcomes [9,15,16] rather than the methodological issues which these studies are commonly intended to investigate. Such studies may fail to detect or address any of the subsequent threats to internal or external validity, and certainly do not have sufficient sophistication to identify trade-offs or potential solutions. Second, the reporting of pilot trials and feasibility studies may be sub-optimum. Given their importance, such studies ought to be carefully designed, with clear aims and objectives to support their own scientific validity in order to appropriately inform decisions regarding any future trial [16,17]. Yet in a recent review of published pilot trials and feasibility studies only 56% of the identified studies reported issues that would be useful in the development of a main trial [15].

In this paper we present an example of a feasibility study, which included a pilot trial, of a complex intervention. We reflect upon the importance of feasibility work and how a better understanding of the processes of developing the definitive trial following a feasibly study or pilot trial might contribute to the debate regarding explanatory and pragmatic trials. We discuss the methodological implications for future feasibility study conduct and reporting. The example relates to a complex intervention for the conservative management of female pelvic organ prolapse. It was hypothesized that undertaking pelvic floor muscle training (PFMT) with a vaginal pessary in place would lead to additional improvements in prolapse symptoms and quality of life for women with prolapse beyond that expected from a pessary alone. PFMT and pessaries are commonly used independently to treat pelvic organ prolapse [18,19]. There is growing level one evidence to support the effectiveness of PFMT [20,21]. In addition, some research suggests that use of a pessary may improve the anatomically defined stage of prolapse [22], and a woman’s symptoms and quality of life [23-25]. However, there is no evidence relating to the combination of these treatments, and whether a combination is more effective for prolapse than treatment with either alone.

Due to the complex nature of PFMT, which involves multiple patient/therapist contacts and a progressive tailored home exercise programme, there was a need to consider evaluation of the PFMT plus pessary intervention in the light of the MRC’s framework for evaluation of complex interventions [2]. Furthermore, while there were some aspects of design which could be confidently determined based on previous research in this clinical area (for example, the primary outcome measure had been developed and tested [26]), there were other aspects of the design for which evidence was lacking, and therefore a feasibility study, including a pilot trial, was deemed necessary prior to undertaking a definitive trial.

The aims of this feasibility study and pilot trial were: to determine the feasibility of conducting an RCT of the effectiveness of a PFMT intervention in addition to a vaginal pessary versus a vaginal pessary alone in improving prolapse symptoms, quality of life, and prolapse severity (including assessing issues of eligibility, checking all components of the intervention work together); and to develop and test the methods for a main trial (including sample size calculations, eligibility, recruitment, consent, randomization, adherence to the intervention, participant retention, logistics of a multicenter randomized controlled trial (RCT)).

The aims of this paper are: to outline the processes we used to explore the best pathway forward from feasibility study and pilot trial to main trial and ‘real-world’ implementation; to describe a process designed to assist researchers in making best use of the findings from their feasibility and pilot work to inform their subsequent decisions regarding a follow-on trial; and to illustrate the use of the process using an exemplar feasibility study, the Pessary Plus Physiotherapy for Pelvic Organ Prolapse (PEPPY) study.

## Methods

### PEPPY feasibility study and pilot trial methods

The research design assessed both issues of feasibility and issues of implementation for the main trial. We assessed feasibility through progress in the pilot trial rather than separately. In hindsight, qualitative methods would have been a useful addition to allow exploration of clear difficulties that subsequently arose.

Four centers were involved in recruiting pilot trial women who were new outpatient attendees at gynecology clinics with symptomatic prolapse and fitted with a pessary. Women were eligible to participate in this study if they were 18 years or older, confirmed to have prolapse of any type, of severity stage I to IV (as measured on vaginal examination using the pelvic organ prolapse quantitation (POP-Q) method, [27]), and were successfully fitted with a pessary which was still in place after 2 weeks. Women were excluded if they had previously had a pessary or been formally taught PFMT, if there were contraindications to either of these treatments, or if they were unable to give informed consent. The women targeted for the pilot trial had been screened for another trial, for which they had been ineligible due to the fact they had a pessary fitted. A patient information leaflet was provided and written informed consent obtained. Ethical approval for the study was obtained from the West of Scotland Research Ethics Committee (REC) 1 on 7 December 2007 (07/S0703/141).

At the appointment when the pessary was fitted, women were told about the study. If agreeable, women were then contacted by the study office to discuss their participation. Consenting participants were randomized into one of two groups: 1) PFMT (delivered by a specialist women’s health physiotherapist at five appointments over 16 weeks) in conjunction with pessary management of their prolapse (intervention group); or 2) pessary management alone (control group). Participants had a nurse appointment 6 months after randomization at which time their pessary was removed for 1 month, and an assessment of vaginal tissues and symptoms was undertaken. At 7 months after randomization, participants attended a review appointment with their gynecologist to have their prolapse re-assessed, the pessary re-fitted if necessary, and other treatment needs discussed. Participants received postal questionnaires at baseline (after pessary fitted but prior to randomization), 6 months post-randomization (prior to seeing the nurse to have their pessary removed), and 7 months post-randomization (prior to the 7-month gynecologist review appointment), and recorded symptoms in a diary for 1 month after removal of the pessary at 6 months. The primary outcome of the pilot trial was prolapse symptoms measured using the pelvic organ prolapse symptom score (POP-SS) [26]. Other secondary prolapse outcomes were: prolapse-related quality of life, prolapse severity (POP-Q) [27], and perceived change in prolapse since pessary fitted. POP-Q assessment at 7 months was undertaken blind to the group allocation and previous POP-Q results.

#### Analysis methods

No systematic guidance on how to categorize and explore issues that have arisen in a feasibility study was identified. However, Shanyinde *et al*. [15] reported 14 issues that need to be evaluated in feasibility studies or pilot trials. Shanyinde *et al*. were not completely clear on how those 14 issues were developed; however, some issues may have arisen from the detailed literature review they had undertaken, and all appear to have a degree of face validity. We used the list of 14 methodological issues (as the best available) to categorize and assess the extent to which each issue was addressed (or not) in this feasibility study with pilot trial. Subsequently, we developed a process to support robust and systematic decision-making in moving from feasibility study to full trial and on to real-world implementation. The feasibility and pilot data from the exemplar study were examined in order to address issues highlighted by using the Shanyinde *et al*. methodological issues as a framework [15], and to test out the newly-developed process. Pilot trial data were analyzed using descriptive statistics, but no statistical testing was undertaken as the study did not aim to draw inferences from the data.

## Results

The results of applying the methodological issues as an analytic framework [15] to the exemplar study findings are presented under each of the 14 items summarized in Table 1.

**Table 1:** Summary of findings against 14 methodological issues for feasibility research

**Methodological issues**	**Findings**	**Evidence**
1. Did the feasibility/pilot study allow a sample size calculation for the main trial?	Achieved even though sample was small	16 out of target of 50 participants achieved in feasibility study
		103 per group indicated for main trial
2. What factors influenced eligibility and what proportion of those approached were eligible?	Ineligibility for randomization was mainly due to participant refusal	31 out of 66 approached were eligible
3. Was recruitment successful?	Recruitment was very difficult. Issues at center, clinician, and participant levels	Centers showed low enthusiasm.
		Clinicians failed to identify participants
		Eligible participants not willing to take part (11/31 withdrew pre-randomization)
4. Did eligible participants consent?	Low conversion to consent	16 (52%) randomized out of 31 eligible participants
5. Were participants successfully randomized and did randomization yield equality in groups?	Worked well	Equal sized groups, well-balanced on the minimization variables
6. Were blinding procedures adequate?	Where used, blinding worked well	Self-reported evidence from gynecologists suggests blinding of POPQ measurement was successful
7. Did participants adhere to the intervention?	Good adherence to PFMT appointments and less so to diaries	Physiotherapy appointments attended: n = 5, 62.5%; n = 4, 12.5%; n = 3, 12.5%; n = 2, 12.5%
		Exercise diaries returned: n = 4, 50%; n = 3, 25%; n = 2, 12.5%; n = 0, 12.5%
8. Was the intervention acceptable to the participants?	Not directly assessed but low numbers recruited suggest some difficulty	15 eligible participants decided not to be randomized once all information was available
9. Was it possible to calculate intervention costs and duration?	Assessed in the linked trial	Cost of PFMT: £170 for mean of 4.2 appointments attended
10. Were outcome assessments completed?	Outcome measures used did assess main areas of interest	See summary of outcome data in Table 2
11. Were outcomes measured those that were the most appropriate outcomes?	Sexual problems questionnaire completion poor	Only 25% of participants completed all items at baseline
12. Was retention to the study good?	Once recruited retention was good	Response rates:
		6-month questionnaire: 100% I, 87.5% C
		7-month questionnaire: 75% I, 37.5% C
		Symptom diary completion: 62.5% I, 37.5% C
		6-month appointment: 87.5% I, 50% C
		7-month appointment: 75% I, 37.5% C
		Agreed to pessary removal: 66.7% I, 33.3% C
13. Were the logistics of running a multicenter trial assessed?	Some centers recruited better than others. The center that recruited well had a dedicated local recruiter	Center 1: 15 participants (dedicated recruiter)
		Center 2: 1 participant
		Center 3: 0 participants
		Center 4: 0 participants
14. Did all components of the protocol work together?	Components had strong synergy	There were no difficulties identified in the various processes and the researcher’s ability to implement them. For example, if participants were recruited, they were easily randomized and their care moved forwards to the appropriate trial arm.

Methodological issues based on Shanyinde *et al*. [15]. C, control; I, intervention; PFMT, pelvic floor muscle training; POP-Q, pelvic organ prolapse quantitation.

### Sample size calculation

The number of participants recruited in the pilot trial was small (n = 16 out of the target of 50 participants); indeed, poor recruitment was a key issue within the study. The sample size for a potential main trial was, however, calculated based on the difference between groups in the primary outcome measure observed at 6 months. It was estimated that a future trial powered to detect the observed difference (3 points on the POP-SS, SD 7) would require 103 participants per group (90% power, 5% level of significance, two-sided). Our work on developing the POP-SS instrument, which is based on women undergoing surgery or PFMT, has given a preliminary estimate of the minimally important change in POP-SS score of 1.5 [28]. Therefore, as women with pessary treatment may be different from those on whom original testing was undertaken, we have chosen a conservative estimate POP-SS score of 3 between groups to ensure the difference is meaningful. Given the small sample size indicated, the question remains: are the observed difference between groups and SD good indicators on which to base such calculations? Our other studies using the POP-SS suggest that the SD differs between populations: treatment naive participants with prolapse in a trial of PFMT, SD 6; participants after surgery for prolapse, SD 8; and participants without prolapse in a longitudinal study of symptoms after childbirth, SD 3.5. This would suggest that the SD observed in this feasibility study was in keeping with that in other groups of participants known to have a prolapse. If the SD was artificially inflated the impact on sample size calculations would be to increase the sample size, thus providing an overestimate of what was needed. Establishing the difference between groups in the primary outcome measure is of course the aim of the main trial and cannot be accurately estimated in a small sample. Examining estimates of effect size from other studies which have measured the same outcome can also help to inform the main trial sample size.

### Eligibility

**Figure 1:**
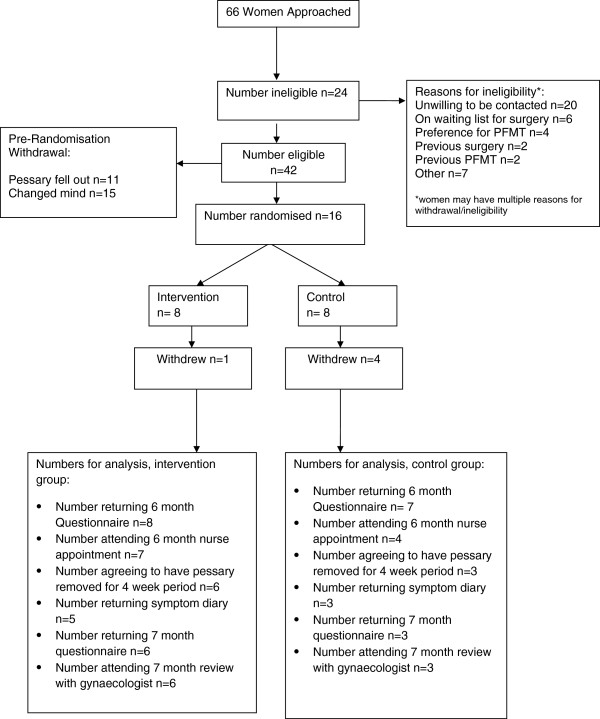
CONSORT diagram.

During the period from 1 February 2008 to 11 February 2010, a total of 66 women were approached to take part (Figure 1, Consolidated Standards of Reporting Trials (CONSORT) diagram). Of these, 24 women were ineligible mainly due to being unwilling to be contacted by the study team after being provided initial information by clinical staff (n = 20). Of the 42 eligible women, 11 did not successfully retain their pessary so became ineligible. A further 15 women either decided not to participate when the study office contacted them to obtain consent, or withdrew once they received paperwork prior to randomization. The remaining 16 participants were randomized, eight to intervention and eight to the control group.

### Recruitment

Recruiting to the study proved very difficult, despite an extended recruitment period and the addition of an extra center. Three centers effectively failed to initiate recruitment. Reasons given by those centers included extremely busy clinics and limited use of pessaries by the recruiting gynecologists. In addition, many women were reluctant to participate as noted above. As women were recruited at a point when the decision to fit a pessary had already been made, this may have affected their motivation to agree to being randomized to receive an additional intervention.

For a considerable number of women, not taking part in the study seemed linked to the fact that they saw the pessary as a permanent solution; if the pessary relieved their symptoms they would keep it in the long-term and there would be no need for additional intervention. One of the hypotheses, however, was that the combination of PFMT and pessary might allow women to become symptom-free, or have reduced symptoms, without the need for a pessary. Thus, to be recruited, women needed to view the pessary as potentially a short-term component of their treatment. The fact that some women did not wish to have their pessary removed at 6 months indicates that this was not always the case.

There was an indication that participants who entered the study were younger and had less severe prolapse than those who did not. This perhaps suggests that the older participants with more severe prolapse favored a pessary on a more permanent basis and therefore were less inclined to take part.

### Consent

Of the 66 women approached by their gynecologist to take part, 20 were unwilling at the outset to be contacted by the study team, and prior to randomization a further 15 women decided not to participate. Thus, over half of the women approached and otherwise eligible chose not to consent to take part. Better counseling of women by clinical staff at study centers may have avoided this problem. In the center where there was a dedicated person undertaking screening, gaining consent was more successful.

### Randomization procedures

Once participants had completed and returned their consent form their group allocation was determined. The research assistant randomized participants into one of the two groups using a randomization programme in Access (Microsoft, Redmond, WA, USA) developed by the Centre for Healthcare Randomized Trials (CHaRT), University of Aberdeen, Aberdeen, UK. Randomization was minimized on center, stage of prolapse (I, II, III, IV), type of pessary fitted (ring or shelf), and whether topical estrogen was being used (yes, no). The processes for achieving randomization worked smoothly.

### Blinding procedures

Blinding of participants and the intervention physiotherapists was not possible given the nature of the intervention. However outcome assessment was mainly through participant-completed questionnaires, thus avoiding assessor bias. Gynecologists were blinded to participants’ group allocation at the 7-month follow-up prolapse review appointment.

### Adherence to intervention

Adherence in terms of replying to study questionnaires was high at baseline and 6 months, but at 7 months was poorer, particularly in the control group. Attendance at the 6-month nurse appointment and the 7-month gynecology review appointment was poor in the control group. Adherence with attending physiotherapy appointments was good, with 75% of participants in the intervention group attending four or five of the planned five appointments, and all except one participant completed the exercise diaries between appointments.

### Acceptability of intervention

Acceptability was not assessed directly but adherence to physiotherapy gives an indication, as does the initial (poor) rates of willingness to participate. It may be that the intervention and the trial processes were acceptable to some participants (those that participated and adhered to the protocols) but not others (those who chose not to take part, perhaps due to being put off by the intervention or the associated trial processes, or by other commitments). Post-randomization withdrawals were common in the control group suggesting that control participants may have been dissatisfied with their allocation.

### Cost and duration of intervention

No economic evaluation was included in the study but cost information was available from a related trial about cost of PFMT [29].

### Outcome assessment

The participants mainly had prolapse involving the anterior vaginal wall, of stage II or III severity. Of the 16 participants randomized, five withdrew post-randomization (one intervention and four control participants) for various reasons; however, four of these participants continued to complete questionnaires. Two participants did not wish their pessary to be removed at the 6-month stage. Seven-month POP-Q data were available for nine participants.

The randomized groups were comparable at baseline with regard to demographic and outcome variables. The mean age of participants who were not randomized was slightly older (70.52 years, SD 13.56, n = 40) compared to those randomized (63.12 years, SD 14.301, n = 16) suggesting a slight selection bias towards younger women participating in the study (t = 1.817, df = 54, *P* = 0.075). Non-randomized participants also tended to have more severe prolapse (stage I, 0%; stage II, 60%; stage III/IV, 40%) compared to randomized participants (chi-squared test = 10.920, df = 2, *P* = 0.004).

The mean POP-SS score was greatest (more frequent symptoms) in both groups at the 7-month time-point, indicating worse symptoms after the pessary had been removed (Table 2). The mean score was higher in the control group compared to the intervention group at 6 months, but the reverse was true at 7 months.

**Table 2:** Summary of primary outcome (POP-SS) and main secondary outcomes at baseline, 6 months, and 7 months

**POP-SS score (scores presented as mean (SD) median, range, n)**^a^	**Intervention**	**Control**
Baseline	4.57 (3.87)	3.00 (2.20)
	3, 2 to 13	3, 1 to 8
	n = 7	n = 8
6 months	5.38 (4.72)	8.57 (9.65)
	4, 1 to 14	4, 1 to 28
	n = 8	n = 7
7 months	15.33 (6.35)	12.67 (9.24)
	19, 8 to 19	18, 2 to 18
	n = 3	n = 3
**Change in POP-Q stage**^b^	n = 6	n = 3
+ 2 stages	0	0
+ 1 stage	2 (33%)	2 (66%)
No change in stage	3 (50%)	1 (33%)
−1 stage	0	0
−2 stages	1 (17%)	0
**How do you feel your prolapse is now compared to when your pessary was first inserted?**		
*6 months*	n = 7	n = 7
Better	5	5
Same	2	1
Worse	0	1
*7 months*	n = 6	n = 3
Better	3	1
Same	2	1
Worse	1	1

^a^POP-SS score: 0 = none, 28 = all symptoms all the time; ^b^a negative value indicates an improvement at 6 months. POP-SS, pelvic organ prolapse symptom score; POP-Q, pelvic organ prolapse quantitation.

Comparable changes in POP-Q stage were observed in the intervention and control group (Table 2). There was no difference between the study groups in terms of the subjective change in prolapse that participants reported in the 6- and 7-month questionnaires (Table 2).

### Selection of most appropriate outcomes

The most appropriate outcomes to use were decided beforehand based on the experience of the researchers in other prolapse studies [26,29-31]. However, response to the sexual symptom questionnaire was poor with some participants finding these questions intrusive.

### Retention

Information on follow-up questionnaire rates is included under the adherence section. Once participants were randomized follow-up was generally good, both in terms of attendance at appointments and completion of questionnaires, suggesting that this part of the methodology would be transferrable to a larger trial. Adherence did appear to be poorer in the control group, and comments from some participants suggested that this was because they considered they were ‘missing out’ due to their allocation to the control group.

### Logistics of multicenter trial

Some centers failed to recruit, suggesting that there may be some difficulty with implementing a multicenter trial. The center that recruited the majority of participants had dedicated local researchers responsible for local trial operationalization and recruitment.

### All components of the protocol work together

Undoubtedly, we gained experience of delivering the various components contained within a protocol that aimed to deliver and evaluate a complex intervention. This was the main strength of running the trial on a small scale beforehand, which was a miniature version of the (then) planned main trial.

## Discussion

Despite some aspects of the pilot study running relatively smoothly (for example, randomization and general adherence), data revealed the existence of a number of feasibility issues in designing and operationalizing a full trial. These included problematic center/clinician engagement, low initial patient willingness to participate, significant drop outs post-consent but pre-randomization, introduction of potential selection bias at recruitment, and low levels of data completion with regard to exercise diaries.

### Methodological choices, challenges, and trade-offs in responding to the feasibility results

Given the issues outlined, we concluded that the trial was not feasible in its current form. The study did suggest that issues such as randomization, blinding, adherence to the intervention, outcome assessment, retention, and all components working together were reasonably non-problematic. In addition, sufficient data was obtained to inform a sample size calculation. However, a number of important changes would also need to be made: we lacked information about the population to whom this intervention may be best targeted towards, mainly in terms of acceptability; despite some helpful studies of pessary use [19,32,33] we lacked information about use of pessaries by clinicians (recruitment); we identified that recruiting post-pessary treatment decision was probably not the best strategy (eligibility/recruitment/consent); we identified the need to change to using dedicated local recruiters (recruitment/consent); contemporary evidence published after the start of the feasibility study also suggested a third arm to the trial should be added (intervention); and one outcome was poorly completed and needed reconsideration (outcomes).

Consideration of the challenges highlighted by the feasibility study revealed two principle methodological challenges: 1) deciding what to change in the light of the pilot study findings (the intervention, the trial design, and/or the clinical context) and the implications of those changes for the explanatory or pragmatic nature of the trial; and 2)a lack of formal or informal guidance on how decision-making in response to pilot trial findings should be made and/or documented. These are explored in turn below.

### How should decisions regarding what to change as a result of the findings of a pilot RCT be made?

**Figure 2:**
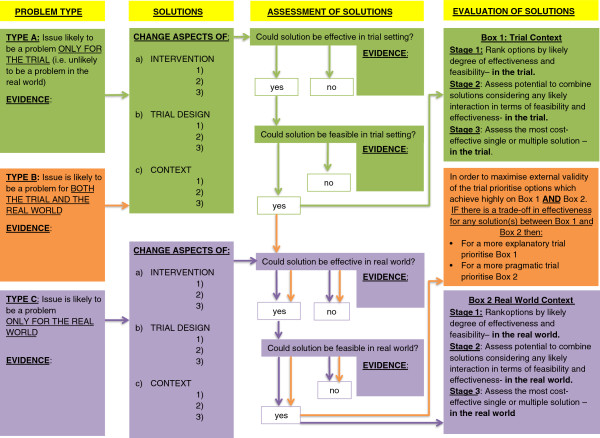
A process for Decision-making after Pilot and feasibility Trials (ADePT).

We identified four potential options in regard to addressing the problems identified: 1) adapting the intervention; 2) adjusting the clinical context within which the intervention would be delivered; 3) amending elements of the trial design; or 4) a combination of any of the former (Figure 2). We soon found that many of the problems identified in the feasibility study could be addressed by targeting any or all of the above. However, further consideration revealed that some solutions may solve the problems for the trial, but would not work subsequently in the ‘real-world’, thereby storing up the problem for later implementation. Below we demonstrate this in regard to recruitment.

If we assume that problems faced around recruitment of participants stemmed largely from a lack of acceptability of the intervention, then a challenge is immediately faced. Given the fundamental nature of the intervention, including its relative simplicity, there were few parameters that are open to adaptation. One might be the use of financial incentives to aid recruitment and even adherence. Certainly, this is gaining increasing popularity in regard to a range of health and illness behaviours [34,35] and is showing some degree of effectiveness, at least within the short-term [36,37]. The decision to use incentives is methodologically acceptable and compatible with an explanatory trial. Demonstrating potential benefit is central to such trials and as such necessitates strong study control and maximizing high patient adherence [13,38]. Indeed, Thorpe *et al*. argued that ‘the more rigorous a trial is in measuring and responding to noncompliance of the study participants, the more explanatory it becomes’ [39], p.54. However, we had originally intended the purpose of the final trial to be largely pragmatic, demonstrating potential real-world benefit and thereby providing helpful information for both policy makers and clinicians in their decision-making processes [40]. In order to achieve this, the benefit and feasibility of the intervention must be demonstrated in real-world conditions. With regard to adherence, Godwin *et al*. [12] suggested that this means that attempts can be made to improve compliance, but these must not go beyond what would be expected in routine practice. We are therefore left with two apparent choices: to use incentives to increase recruitment and adherence but provide results from a final trial that are inapplicable to the real-world, or not to use incentives and to conduct a pragmatic trial that is likely to show effectiveness despite potential benefits (if only participants would adhere).

Despite this apparent tension, we would argue a third option exists: to redefine incentives as an added parameter of the intervention rather than representing a change to context/routine clinical practice. This means increasing the complexity of the intervention based on empirical data within the feasibility study; the intervention now has two components: pessary and incentive. If defined as such then the benefits of external validity (generalizability), the strength of the pragmatic design, is retained as the inference from the trial and that benefit stems from pessary use plus incentives within routine clinical practice rather than pessary alone. The inclusion of additional intervention parameters in order to address contextual weaknesses is not new, and may more commonly be included as the educational component required for clinicians to fully engage with and deliver the intervention [41]. If the added parameter is financial, it may be beneficial to consider economic evaluation within feasibility work, particularly in the context of pragmatic trials.

Pragmatic trials are known to frequently test intervention ‘strategies’ rather than simple devices per se, allowing greater clinician and patient discretion with regard to the adoption and use of the intervention [38]. However, the current literature on explanatory versus pragmatic trials has not adequately addressed the question as to how or when such a strategy is developed or decided upon, nor has the literature on feasibility and pilot studies. The 2000 version of the MRC framework for complex interventions suggests a linear process in which feasibility and pilot testing are conducted after a period of modeling and intervention definition, thus the intervention is largely defined [42,43]. This study, however, suggests that the process of intervention development and definition may well continue post-feasibility and pilot. This is certainly more akin to the less linear and more iterative process included in the updated 2008 framework [1].

### Can guidance to support evidence-based and documented decision-making in response to pilot RCT findings be produced?

The final methodological problem that we faced was the lack of any detailed guidance to support the complex decision-making required, and there did not appear to be any requirement to make the alternative amendments, their consequences, or the rationale for the final choice explicit to external audiences. At the heart of any such guidance must be the assurance that the endpoint will be a trial design that is fit for purpose [13].

Ultimately, we would argue that by the introduction of greater rigor at this stage of the intervention and trial design process, the final utility, through real-world effectiveness and likelihood of real-world implementation, will be significantly enhanced. Several authors have highlighted that funders have historically preferred the rigor and internal validity offered by explanatory trials [44]. Certainly, this is the case for pharmaceutical companies where the explanatory trials are likely to show larger effect sizes for more narrowly defined patients within more controlled circumstances. However, the move towards more pragmatic trials has been largely fuelled by an increasing willingness to apply RCT methods to more complex interventions (which naturally tend to be less well-defined [45], more flexible [46], and more context-dependent [45]), and a recognized need to provide information of greater utility to policy-makers, clinicians, and patients [40,47,48]. If utility to stakeholders is a likely criterion of success then it would seem that their perspectives would need to be taken into account when assessing the varied strategies that might be adopted in response to problems highlighted by feasibility studies. Again, this suggests not only that current guidance on criteria that feasibility and pilot studies should address might benefit from expansion, but also the process of suggestions and recommendations about decision-making processes as well. It is this gap that A process for Decision-making after Pilot and feasibility Trials (ADePT) seeks to fill.

### ADePT

Given the tensions highlighted, we sought to establish a systematic means of supporting and documenting future decision-making in this area. Consequently, we developed the ADePT process to: 1) encourage the systematic identification and appraisal of problems and potential solutions; 2) improve the transparency of decision-making processes; and 3) reveal the tensions that exist between pragmatic and explanatory choices. The process is shown in Figure 2, with a worked example addressing the issue of acceptability of the intervention to the PEPPY study shown in Additional file 1. The ADePT process includes 3 key steps.

#### Step 1: Deciding on the type of problem experienced and the evidence to support that allocation

Three types of problems are possible: type A, the issue is likely to be a problem only for the trial (that is, unlikely to be a problem in the real-world); type B, the issue is likely to be a problem for both the trial and the real-world; and type C, the issue is likely to be a problem only for the real-world.

For each type, it is necessary to expose the evidence for why the problem exists and what type of problem it is. In the example (Additional file 1), evidence from the failure to achieve recruitment and from methodological papers suggest that acceptability of the intervention (the pessary) was a problem. Furthermore, the intervention (pessary) is an existent clinical tool and not amendable to adaptation; therefore, the acceptability issue was highly likely to be a problem for the trial and also a problem for the real-world implementation. This typology is crucial for later decision-making in order to facilitate the choice of a solution that will not only provide a short-term solution for the trial but also, if necessary, a longer-term solution that would be possible to implement in the real-world.

#### Step 2: Identifying the range of potential solutions and the evidence to support those solutions

Potential solutions may apply to the intervention, the trial design, or the clinical context. Solutions can be identified in various ways, including: literature searching; debate/ brainstorming within the research team (ideally including lay representation); and, if available, through analysis of feasibility study data. The documentation of the identified solutions lays the process open to scrutiny, and hence debate and transparency. Depending on the type of problem (type A, B, or C), each solution will need to be assessed in regard to its ability to address or ameliorate the problem in the trial, or real-world clinical context, or both. The assessment of each option should be in terms of: 1) the potential effectiveness of the solution; and 2) the potential feasibility of the solution. Evidence to support each assessment should, ideally, be provided. In the documented example (Additional file 1) we assessed solutions through searching the research literature (focusing on methodological reports and experiences reported within clinical trials), and focused discussions with colleagues who had experience of similar issues and/or potential solutions.

#### Step 3: The assessment of best options

Once all options have been appraised then a final decision should be made. For type A and type C solutions, a simple ranking and assessment as to whether a single or combined solution might be best is likely to suffice; this may also incorporate an assessment of associated costs. For type B problems, a final assessment is likely to be more complex. It is possible that a solution may be identified that is judged to work extremely well in both the trial and real-world, in which case this should be chosen as this option will effectively minimize the tension between internal and external validity – the explanatory trial becomes increasingly synonymous with the pragmatic trial. However, we anticipate that in the majority of cases a judgment may have been made that some solutions may work extremely well within the trial but will work less well within the real-world (or vice versa). In this situation the research team and others may be forced to choose where on the continuum of explanatory to pragmatic [39] they wish to place the trial, and justify this.

We would acknowledge that obtaining robust evidence to support choices and trade-offs at each of these stages is likely to prove difficult. However, decisions will have to be made even if only on tentative evidence or argument. In other areas facing this dilemma, for example, the creation of clinical guidelines, forms of consensus method have been effectively employed [49]. It is possible that this could be explicitly adopted to improve the transparency and rigor of stakeholder decision makers within feasibility studies. Indeed, while Godwin [12] and others have highlighted the frequent trade-off between internal and external validity, it is possible that the inclusion of wider stakeholders in the generation and assessment of solutions may more effectively identify ways of achieving both, or at least minimizing trade-off, for any particular intervention and trial. Documenting and exposing the decision-making process becomes even more important when the evidence to support decision-making is weak, because laying the process bare promotes transparency, robustness, and exposure of the trade-offs made between a more explanatory and a more pragmatic design.

## Conclusions

This feasibility and pilot study successfully gained knowledge about the process and implementation of the proposed main trial. It has also identified the gaps in our knowledge that require to be filled prior to progressing to a main trial. We have undertaken a further survey of professional practice to supplement the study [50] and plan a small qualitative study to explore acceptability to participants. Although the trial is not feasible in the tested form, some adaptations and the further studies will allow us to progress forwards to a stronger main trial. We advocate the use of Shanyinde *et al.*’s work to structure discussion about what has been, or could be learned, from a feasibility study and where the gaps remain [15], and testing the ADePT process to support systematic and transparent identification of issues and solutions for the design of future trials. Our on-going work with ADePT will explore its usefulness when developing an intervention and designing the trial to test it. The worked example and the identification of trade-offs between previously unacknowledged options have raised the question as to whether a pilot RCT should be conducted to assess the feasibility of an explanatory/pragmatic trial, or to decide whether it should be explanatory or pragmatic. Further consideration of this issue is recommended.

## Abbreviations

ADePT: A process for Decision-making after Pilot and feasibility Trials; CHaRT: Centre for Healthcare Randomized Trials; CONSORT: Consolidated Standards of Reporting Trials; df: degrees of freedom; MRC: Medical Research Council; PEPPY: PEssary Plus Physiotherapy for Pelvic Organ Prolapse; PFMT: pelvic floor muscle training; POP-Q: pelvic organ prolapse quantitation; POP-SS: pelvic organ prolapse symptom score; RCT: randomized controlled trial; REC: Research ethics committee.

## Competing interests

The authors declare that they have no competing interests.

## Authors’ contributions

SH was chief investigator, and CG, SP, and LS were co-investigators on the PEPPY trial. JL was the research assistant on the PEPPY trial. CB and SH undertook the analysis using the Shanyinde *et al*. [15] methodological issues as a framework. BW, CB, and SH developed the ADePT process. CB, BW, and SH drafted the manuscript. All authors commented on the manuscript.

## Supplementary Material

Additional file 1 **Worked example using ADePT **[51]**-**[69]**.** Click here for file
