# Supplementary material for: A process for Decision-making after Pilot and feasibility Trials (ADePT): development following a feasibility study of a complex intervention for pelvic organ prolapse
Source: Trials. 2013 Oct 25;14:353. doi: 10.1186/1745-6215-14-353 (PMC3819659; doi:10.1186/1745-6215-14-353)
Supplement: Additional file 1 — Worked example using ADePT [51]-[69]. [file 1745-6215-14-353-S1.pdf]

PROBLEM TYPE

TYPE B: There was a problem with acceptability of the intervention to women.

EVIDENCE:

1. 20 of 66 women asked refused to be approached by the study team (31.7%).
2. 15 of 31 eligible women decided not to take part prior to randomisation (48.3%).
3. Younger women and those with less severe prolapse were more likely to be recruited (self-selection bias).
4. Acceptability may have been influenced by women being approached after decision to use a pessary was made (women not in equipoise).
5. Acceptability may have been influenced by clinicians not being in equipoise.

All of the above may influence treatment decision-making in the trial and in the real world

→

SOLUTIONS

CHANGE ASPECTS OF:

a) INTERVENTION

1) Decrease burden of intervention to women by decreasing number of appointments or offering alternatives to face-to-face appointments (e.g. phone calls).
2) Improve the education offered as part of the intervention, particularly to target equipoise in evidence.

b) TRIAL DESIGN

1) Change the timing of when the women are approached (to pre treatment counselling and pre pessary decision).

c) CONTEXT

1) Offer incentives to women
2) Offer incentives to clinicians (centres)

ASSESSMENT OF SOLUTIONS (INTERVENTION AND TRIAL DESIGN)

Could solution a1 be effective in trial setting?

EVIDENCE: Studies suggest that to be effective 6 PFMT sessions are needed and 16 weeks exercise are needed for muscle change [20,51]. There is some evidence that technologically based input can influence behaviour change [52-54]. A recent Cochrane review found trials that used alternatives to direct HCP contact for PFMT with little evidence of effect [55]

yes

Could solution be feasible in trial setting?

EVIDENCE: no clinical value in decreasing PMFT timeline or number of appointments as this may link to a decrease in efficacy. Other forms of motivation that have less participant burden may be useful but currently lack evidence.

Yes – list in box 1

Could solution **a1** be effective in real world?

EVIDENCE: Alternatives to HCP direct contact are possible if effective.

yes

Could solution be feasible in real world?

EVIDENCE: Yes, if cost-effective.

yes

List in box 2

Could solution a2 be effective in trial setting?

EVIDENCE: Both patients and health professionals not being in equipoise are identified as barriers to trial recruitment [56-60]; education is a possible solution. However evidence of effectiveness of education on changing equipoise specifically is lacking. Evidence of education changing professional practice does exist [61] and there is evidence of educational effectiveness on patient outcomes [62,63]

yes

Could solution be feasible in trial setting?

EVIDENCE: Yes, embedded within intervention as part of trial instigation.

Yes – List in box 1

Could solution **a2** be effective in real world?

EVIDENCE: Educational interventions tested in real world settings.

yes

Could solution be feasible in real world?

EVIDENCE: Continuing education required in professional practice; patient education used routinely.

yes

List in box 2

Could solution b1 be effective in trial setting?

EVIDENCE: patients not being in equipoise is identified as a barrier to trial recruitment [56-58].

yes

Could solution be feasible in trial setting?

EVIDENCE: If provider in equipoise, patients receiving counselling about all options would be akin to ethical practice.

Yes list in box 1

Could solution be effective in real world?

EVIDENCE: If intervention is effective would be one option within normal treatment counselling

yes

Could solution be feasible in real world?

EVIDENCE: as part of normal practice.

yes

List in box 2

ASSESSMENT OF SOLUTIONS (CONTEXT)

Could solution **c1** be effective in trial setting?

EVIDENCE: There is some evidence to suggest that patient incentives may increase service uptake, with the possibility (although the evidence is unclear) of some behaviour change in the short term [36, 64, 65].

yes

Could solution be feasible in trial setting?

EVIDENCE: Yes, has been used in other studies within a health care context and some success in increasing uptake.

Yes – list in box 1

Could solution **c1** be effective in real world?

EVIDENCE: Incentives are being accepted in health care as a valid part of an intervention that aims to change behaviour to achieve health gain. Translated to the current study, gain could be in relation to a woman's symptomatic improvement or improved Quality of Life (QoL).

yes

Could solution be feasible in real world?

EVIDENCE: Yes, although still uncommon, incentives are starting to be used in service delivery. Evidence of cost-effectiveness would be needed

yes

List in box 2

Could solution **c2** be effective in trial setting?

EVIDENCE: : Some suggestion that incentives to professionals/ services improves uptake by patients [66-68] but also some evidence of no effect [69].

yes

Could solution be feasible in trial setting?

EVIDENCE: Yes, has worked in other trials.

Yes – list in box 1

Could solution **c2** be effective in real world?

EVIDENCE: Trials tested in real world contexts with evidence of improved uptake.

yes

Could solution be feasible in real world?

EVIDENCE: Yes, service has structures that allow payment for services **BUT** would need evidence of cost-effectiveness

yes

List in box 2

EVALUATION OF SOLUTIONS

BOX 1: OPTIONS THAT SHOULD WORK IN TRIAL CONTEXT

Stage 1: Options (ranked by likely feasibility & effectiveness):

1. Education for equipoise
2. Pre-treatment counselling
3. Incentives for Health Care Professionals (HCP)
4. Incentives for women
5. Decrease burden for women

Stage 2: Potential to combine solutions
Equipoise is important to maximise recruitment and ensure appropriate shared decision-making. Pre-treatment counselling is akin to current best practice. HCPs (or their employers) are commonly given incentives that are shown to maximise health gain. Giving women incentives in the trial may increase the numbers of women who take part (and hence maximise the chance to prove efficacy). Decreasing patient burden may increase patient recruitment.

Stage 3: Most Cost Effective Solution
HCPs and women in equipoise is important and central to allowing participant recruitment to take place. Incentives and treatment counselling are easily incorporated into trial design. Hence a multi-level strategy is possible.

→

BOX 2 OPTIONS THAT SHOULD WORK IN REAL WORLD CONTEXT

Stage 1: Options (ranked by likely feasibility & effectiveness):

1. Pre-treatment counselling (good practice)
2. Decreasing patient burden (patient preference and service efficiency)
3. Education (part of CPD/ routine patient care)
4. Professional incentives (normal use)
5. Patient incentives (becoming more accepted)

Stage 2: Potential to combine Solutions
Treatment counselling is a feature of best practice so important to incorporate; Decreasing patient burden may improve efficiency of service use; education is routinely used in practice for professionals and patients and part of developing good practice; professional incentives are useful if there is evidence of cost effectiveness and patient incentives have some evidence to support use.

Stage 3: Most Cost Effective Solution
As in box 1 multiple concurrent solutions are possible.

→

BOX 3: FINAL ASSESSMENT OF OPTIONS AND TOLERANCE OF TRADE-OFF BETWEEN EXPLANATORY & PRAGMATIC TRIAL

This is a PRAGMATIC TRIAL therefore box 2 is higher priority than box 1.

Offering women all available treatment options is akin to best practice and therefore moving the point of study introduction to the point of treatment choice will fit in with current best practice. Decreasing burden for patients is positive in itself but may also offer service efficiency. Education for the trial and onwards to real world implementation is part of routine practice and also commonly used with patients (if effective). As clinical incentives are possible in both trial and real world this appears to be a useful solution to include.
